# Case report: HLA-haploidentical HSCT rescued with donor lymphocytes infusions in a patient with X-linked chronic granulomatous disease

**DOI:** 10.3389/fimmu.2023.1042650

**Published:** 2023-02-16

**Authors:** Julia Scheiermann, Annette Künkele, Arend von Stackelberg, Angelika Eggert, Peter Lang, Felix Zirngibl, Luise Martin, Johannes Hubertus Schulte, Horst von Bernuth

**Affiliations:** ^1^ Charité - Universitätsmedizin Berlin, Corporate Member of Freie Universität Berlin, Humboldt-Universität zu Berlin, Department of Pediatric Hematology, Oncology and Stem Cell Transplantation, University Hospital Center, Berlin, Germany; ^2^ Berlin Institute of Health at Charité – Universitätsmedizin Berlin, Berlin, Germany; ^3^ German Cancer Consortium [Deutsches Konsortium für Transnationale Krebsforschung (DKTK)], Berlin, Germany; ^4^ German Cancer Research Center [Deutsches Krebsforschungszentrum (DKFZ)], Heidelberg, Germany; ^5^ Department of Pediatric Hematology and Oncology, University Hospital, Tübingen, Germany; ^6^ Charité - Universitätsmedizin Berlin, Corporate Member of Freie Universität Berlin, Humboldt-Universität zu Berlin, Department of Pediatric Respiratory Medicine, Immunology and Critical Care Medicine, University Hospital Center, Berlin, Germany; ^7^ Department of Immunology, Labor Berlin GmbH, Berlin, Germany; ^8^ Charité - Universitätsmedizin Berlin, Corporate Member of Freie Universität Berlin, Humboldt-Universität zu Berlin, and Berlin Institute of Health (BIH), Berlin-Brandenburg Center for Regenerative Therapies (BCRT), Berlin, Germany

**Keywords:** chronic granulomatous disease, HLA-haploidentical hematopoietic stem cell transplantation, TCR alpha/beta+/CD19+ depleted peripheral blood HSCT, donor lymphocyte infusion (DLI), graft verses host disease

## Abstract

Chronic granulomatous disease is an inborn error of immunity due to disrupted function of the nicotinamide adenine dinucleotide phosphate (NADPH) oxidase complex. This results in impaired respiratory burst of phagocytes and insufficient killing of bacteria and fungi. Patients with chronic granulomatous disease are at increased risk for infections, autoinflammation and autoimmunity. Allogeneic hematopoietic stem cell transplantation (HSCT) is the only widely available curative therapy. While HSCT from human leukocyte antigen (HLA) matched siblings or unrelated donors are standard of care, transplantation from HLA-haploidentical donors or gene therapy are considered alternative options. We describe a 14-month-old male with X-linked chronic granulomatous disease who underwent a paternal HLA-haploidentical HSCT using T-cell receptor (TCR) alpha/beta^+^/CD19^+^ depleted peripheral blood stem cells followed by mycophenolate graft versus host disease prophylaxis. Decreasing donor fraction of CD3^+^ T cells was overcome by repeated infusions of donor lymphocytes from the paternal HLA-haploidentical donor. The patient achieved normalized respiratory burst and full donor chimerism. He remained disease-free off any antibiotic prophylaxis for more than three years after HLA-haploidentical HSCT. In patients with x-linked chronic granulomatous disease without a matched donor paternal HLA-haploidentical HSCT is a treatment option worth to consider. Administration of donor lymphocytes can prevent imminent graft failure.

## Introduction

Chronic granulomatous disease (CGD) is an inborn inborn error of immunity (IEI) characterized by a severely impaired or absent respiratory burst in all phagocytes. Pathogenic variants in one of five genes coding for subunits of the nicotinamide adenine dinucleotide phosphate (NADPH) oxidase complex have been identified. Biallelic defects in *CYBA, NCF1, NCF2* and *CYBC1* (encoding for p22*^phox^*, p47*^phox^*, p67*^phox^* and Eros) cause autosomal recessive CGD (AR-CGD), while mutations in *CYBB* (encoding for the catalytic domain gp91*^phox^*) cause XL-CGD (1). Of note, biallelic pathogenic mutations in *NCF4* cause a related, but distinct disease (2). Malfunctional NADPH oxidase predisposes patients to infections with catalase positive bacteria such as *Staphylococcus aureus*, *Burkholderia cepatia*, *Serratia marcescens*, and fungi such as *Aspergillus* spp. and other molds (3). Residual superoxide production correlates with longer overall survival (OS) (4). Additionally, autoinflammation and autoimmunity with or without granuloma formation can occur potentially in any organ, most often in the bladder and gastrointestinal tract. Although the frequency of infections can be significantly reduced with the prophylactic use of antimicrobials (e.g. trimethoprim-sulfamethoxazole, TMP-SMX), antifungals (e.g. itraconazole or posaconazole) and interferon-γ (INF-γ), overall survival begins to decline in the second decade of life (3). Consequently, the use of allogeneic HSCT in younger patients, preferentially those younger than 8 years of age, improves overall survival (3). In addition, improving medical care and safer conditioning protocols with reduced toxicity conditioning (RTC) were established enabling HSCT for adult CGD patients who have been suffering from fungal infections and/or autoinflammation prior to HSCT (5). If a HLA-matched donor is not available for XL-CGD patients, paternal haplo-HSCT can be used.

However, non-engraftment occurs in a considerable portion of patients with CGD (3). Administration of donor lymphocyte infusions (DLI) is an established immunotherapeutic treatment in patients, who underwent haploidentical-HSCT (haplo-HSCT) and present with mixed donor chimerism (6). However, experience of DLI application for children transplanted for the treatment of CGD is still scarce. Pediatric IEI patients receiving DLI after HSCT responded to the treatment by increasing donor chimerism more effectively in matched unrelated donors. In approximately 80% of patients DLI could prevent graft rejection. Moreover, there is concern, that administration of DLI may induce or aggravate GVHD. Published literature reports acute and chronic GVHD occurrence in 10% to 60%, yet GVHD was mostly rather mild (grade I-II) and effected mostly the skin (7–11). Here we report on a 14-month-old boy with XL-CGD who was treated with paternal haplo-HSCT followed by eight DLI administrations.

## Materials and methods

Informed consent for haplo-HSCT was obtained from the patient’s parents in accordance with local and European Society for Blood and Marrow Transplantation (EBMT) guidelines. Before submission of the manuscript, additional informed consent for publication was obtained from both parents. The day of engraftment was defined as the first of three consecutive days without transfusion and with absolute neutrophil counts (ANC) > 500/μl. The patient underwent weekly polymerase chain reaction (PCR) analysis of blood to detect adenovirus (ADV), Epstein-Barr virus (EBV), and cytomegalovirus (CMV). Acute GVHD was assessed using the modified Seattle Glucksberg criteria (12). Chronic GVHD was scored according to the National Institutes of Health (NIH) criteria (13). Chimerism was monitored in bone marrow and peripheral blood specimens to assess donor cell engraftment. Donor chimerism was measured by labelling blood with anti-CD3 and anti-CD34 micro beads and cell lines were separated using an autoMACS automated benchtop magnetic cell sorter (Miltenyi Biotec). Separated cells were assayed using short tandem repeats *via* multiplex PCR and capillary array electrophoresis (ABI Prism 3100, ThermoFischer). The neutrophil respiratory burst was performed on fresh whole blood samples with Phagoburst kit (DB biosciences) according to manufacturer’s instructions.

## Case description

We describe a 14-month-old male patient diagnosed with XL-CGD (mutation *CYBB* c.175 T>C), who was successfully transplanted with peripheral blood stem cells (PBMC) from his HLA-haploidentical father. The patient was diagnosed with XL- CGD after birth upon a history of CGD in his maternal uncle and his mother being a carrier for the same pathogenic mutation in *CYBB* as identified in her brother.

He received oral itraconazole (4 - 6 mg/kg/d) and cefuroxime (20 mg/kg/d) in the first 3 months of life. The latter was switched to TMP-SMX (4 - 6 mg/kg/d TMP component) thereafter. By the end of the first year of life the patient had developed mild CGD colitis. On pre-HSCT examination, ultrasound showed increased bowel wall thickness. Histology revealed follicular lymphatic hyperplasia and mild eosinophilia in tissue biopsies from caecum, colon, sigma and rectum in line with CGD colitis.

**Figure 1:**
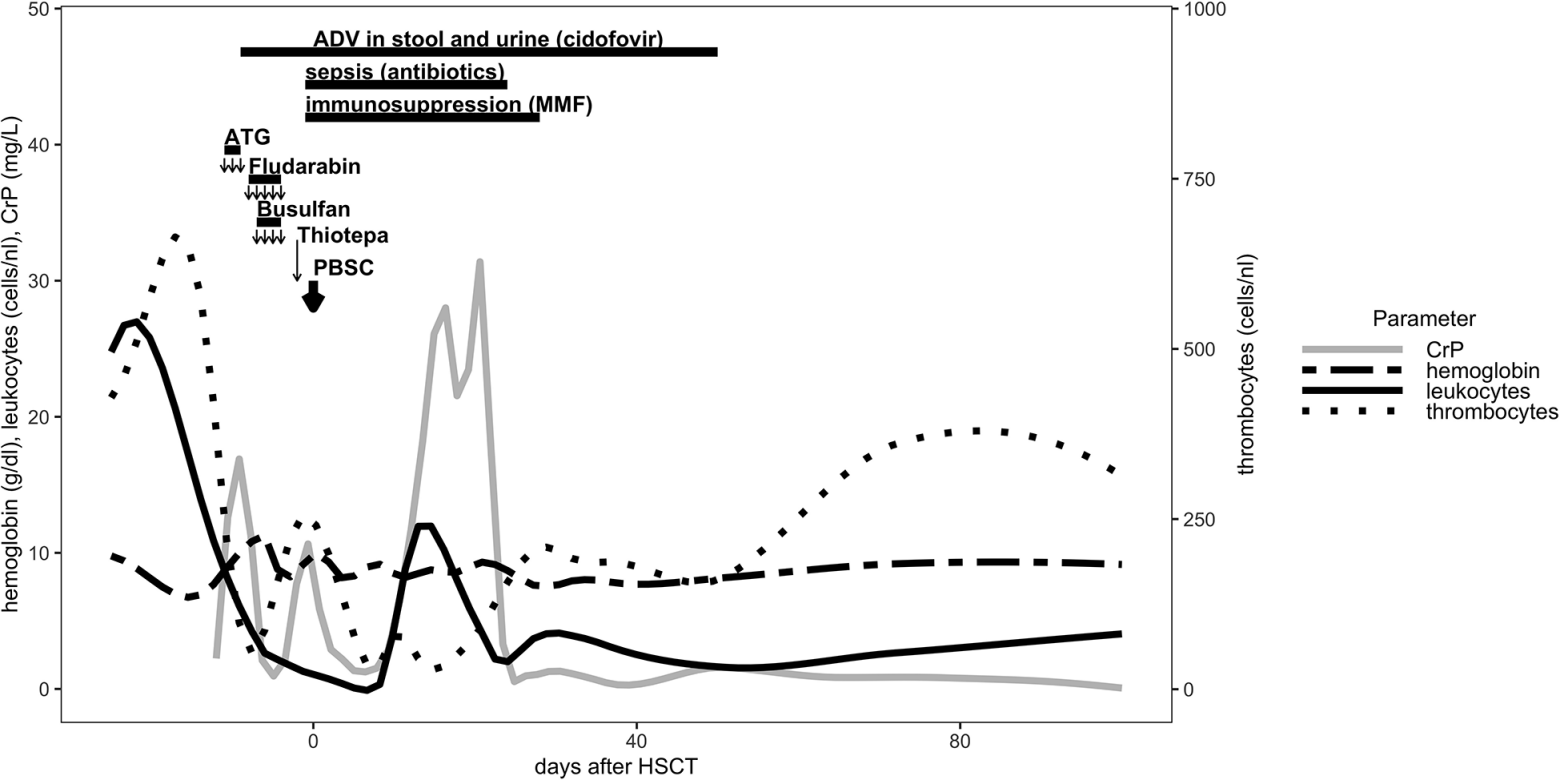
Conditioning regiment, immunosuppression and cellular reconstitution are shown for the first 100 days post HLA-haploidentical HSCT.

The patient underwent a myeloablative conditioning (MAC) regimen at the age of 14 months after a course of antibiotic treatment using cefotaxime and metronidazole from day - 12 to day - 9. Due to CGD colitis, the patient received additional methylprednisolone treatment (20 mg/kg/d) from day - 11 to day - 9. Levetiracetam (20 mg/kg/d) was administered form day - 9 through day + 5 for seizure prophylaxis. The conditioning regimen consisted of rabbit anti-thymocyte globulin (ATG, Grafalon) 20 mg/kg/d (day - 11 to day - 9), fludarabine 30 mg/m^2^/d (day - 8 to day - 4), and thiotepa 10 mg/kg/d on day - 2. Busulfan was given from day - 7 to day - 4 with therapeutic drug monitoring and a final myeloablative cumulative dosage of 174 mg (18.9 mg/kg) and total area under the curve (AUC) of 93553 ng x h/ml. Peripheral blood stem cell graft infusion from the patient’s father was administered on day 0 after TCR alpha/beta^+^/CD19^+^ depletion (**Figure 1**). The cell product contained total nucleated cells 3.35 x 10^8^ cells/kg, with CD34^+^, 53.96 x 10^6^ cells/kg; CD3^+^ 53.28 x 10^6^ cells/kg; TCR alpha/beta^+^ 12.31 x 10^3^ cells/kg; and TCR gamma/delta^+^ 52.27 x 10^6^ cells/kg.

Granulocyte colony stimulating factor (GCSF) (5 μg/kg/d) was administered from day + 5 for 6 consecutive days. Full engraftment occurred on day + 13. The patient received immunosuppressive therapy with mocophenolate mofetil (MMF) at the dosage of 2 x 600 mg/m^2^/d as GVHD prophylaxis from day + 1 until day + 28 (**Figure 1**). During the course of conditioning regimen, the patient developed fever and sepsis with *Serratia marcescens* on day - 1 and received antimicrobial therapy through day + 24. Adenovirus was isolated day – 9 in the stool and urine samples and was treated with cidofovir until day + 50 (**Figure 1**). The prophylactic antifungal treatment with liposomal amphotericin B was administered intravenously starting on day + 1 and was replaced by posaconazol p.o. at day + 30. At day + 14 the patient developed a veno-occlusive disease/sinusoidal obstruction syndrome (VOD/SOS) under prophylactic intravenous heparin according to European society for blood and marrow transplantation (EBMT) diagnostic criteria (14), which was treated with defibrotide (starting at 40 mg/kg/d and escalating to 60 mg/kg/d) from day + 15 until day + 33. Aciclovir and posaconazol were continued prophylactically until day + 251 after HSCT.

**Figure 2:**
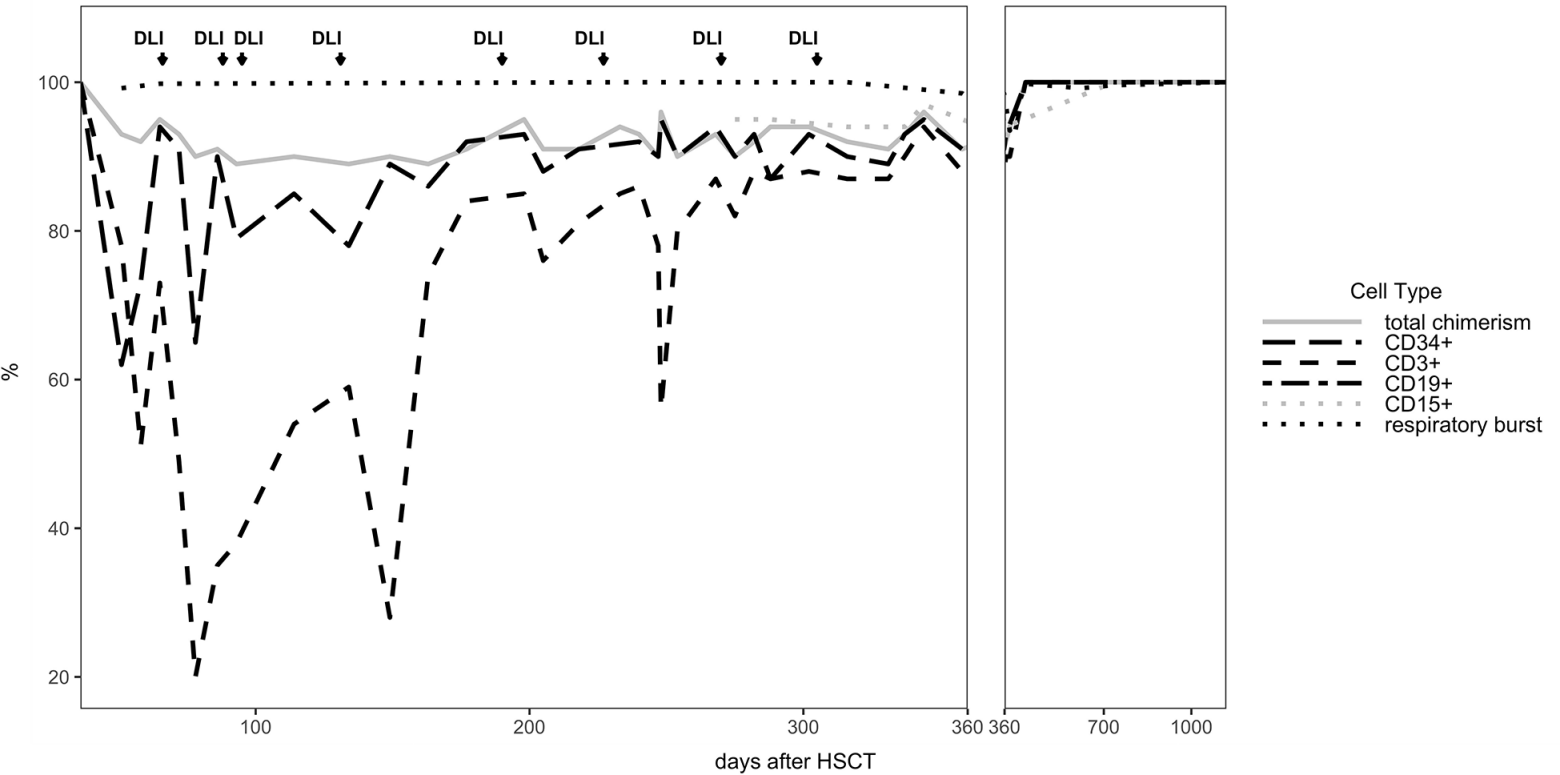
Donor Chimerism in peripheral blood for CD34^+^, CD3^+^, total cells (total chimerism) and respiratory burst in patient’s neutrophils measured by DHR test are shown for the entire follow up time of 3 years.

**Figure 3:**
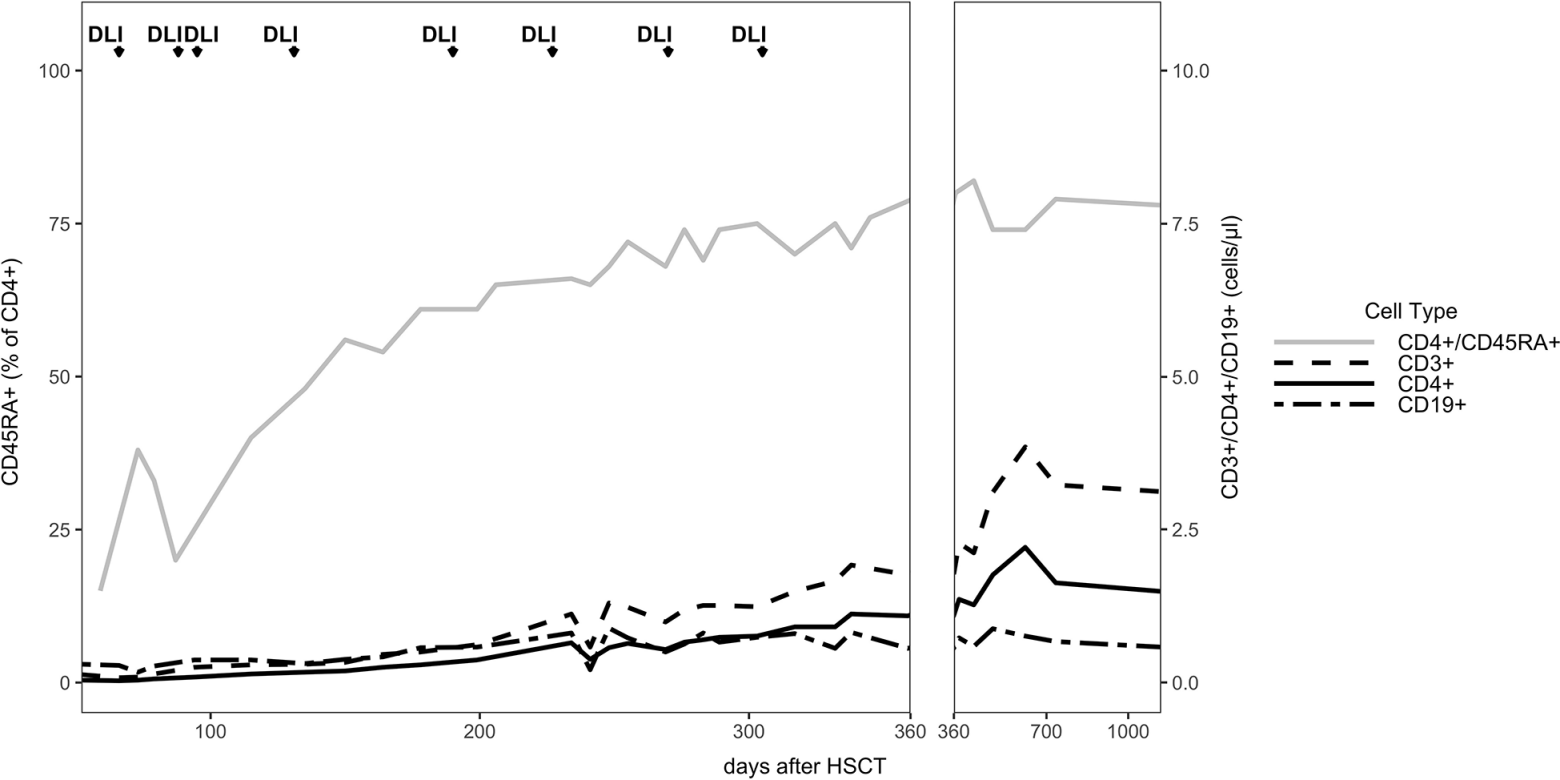
Immune reconstitution: The absolute cell numbers per μl are shown for the entire follow up time of 3 years. The CD45RA+ (CCR7+) percentage of the CD4^+^ cells are shown.

The patient developed mixed donor chimerism beginning at day + 52 with the lowest values for CD3^+^ chimerism of 20% on day + 80 and total chimerism of 89% on day + 95 after HSCT. In parallel counts of CD3^+^ T-cell in peripheral blood also declined, whereas the respiratory burst of patient’s granulocytes, measured by Dihydrorhodamine (DHR) assays upon stimulation with *E. coli* PMA continuously exhibited a normal production of H_2_O_2_ (**Figures 2**, **3**). In total, eight DLI were administered for rescue of the decreasing donor chimerism starting with a cell number of 2.5 x 10^4^ CD3^+^ cells/kg and increasing up to 30 x 10^4^ CD3^+^ cells/kg of patient’s body weight between days + 66 and + 329 (**Figures 2**, **3**; **Table 1**). Peripheral T cell numbers as well as donor chimerism rose post DLI. This was particularly observed after the first four DLI, whereas the following four DLI had a less obvious effect. The patient`s ADV infection neither effected the chimerism nor the number of peripheral T cells. No signs of GVHD occurred after the administration of the DLI. 14 months post transplantation the patient achieved complete donor chimerism which remained stable thereafter. No acute or chronic GVHD developed. Four years after haplo-HSCT our patient is cured from CGD and off any treatment.

**Table 1:** DLI characteristics: dates and dosages of administered CD3^+^ cells are shown.

DLI	day post HSCT	dosage (CD3^+^/kg of body weight)
1	66	25000
2	88	50000
3	95	30000
4	131	30000
5	190	100000
6	233	300000
7	270	100000
8	305	300000

## Discussion

The introduction of safer protocols and effective GVHD prophylaxis has improved the overall survival of CGD patients receiving allogeneic HSCT (5). This seems particularly the case, if CGD patients are transplanted at young age, ≤ 8 years while organ functions are fully retained and severe infectious and autoinflammatory complications have not yet occurred (3). Yet, matched related or unrelated donors are not available for all patients with CGD. Gene therapy is yet not widely available and must still be regarded as rather experimental and prone to serious complications (15). Along this line, haplo-HSCT seems the more evident option for children with CGD in the absence of a matched unrelated donor. Moreover, haplo-HSCT became an attractive option because most patients with CGD still have living parents who can perform as a possible donor. In XL-CGD patients’ mothers are often carriers for a pathogenic mutation in *CYBB*. As this heterozygous state may exhibit skewed lyonisation later in life, only paternal haplo-HSCT is a reasonable option in XL-CGD.

We here report the 22^nd^ published successful haplo-HSCT in CGD (16–29) and the 11^th^ published patient transplanted using a TCR alpha/beta^+^/CD19^+^ depleted PBSC graft (19, 22, 24, 25, 29) (**Supplementary Table 1**). The first haplo-HSCT described by Kikuta et al. in 2006 was performed in a 2-year-old using RIC conditioning, however, that patient developed graft failure on day + 77 and needed a second transplant from a MUD. The use of HLA-haploidentical donors historically entailed an increased risk of graft rejection. To our knowledge, we report the first successful HLA-haploidentical HSCT, who showed mixed chimerism with CD3^+^ T cell donor chimerism as low as 20% at day + 80, as sign of imminent graft failure, in which the graft could be rescued with repeated DLI infusions from his paternal donor. By the time of 14 months after haplo-HSCT stable donor chimerism was observed in this patient (**Table 1**).

Until today only few pediatric case series could show beneficial use of DLI cellular immunotherapy in settings of graft rejection after HSCT in IEI (7–11). Our case shows how important it is to monitor the lineage specific chimerism after haplo-HSCT in CGD to initiate DLI administrations. Similarly, in patients with hemoglobinopathies mixed donor chimerism and graft rejection post HSCT, regardless of the donor source, are frequently observed and may also be rescued by DLI (9, 30, 31). Of note, until this day no definite recommendation from the EBMT working party exists regarding the use of DLI after haplo-HSCT (6). Our report enforces the notion that if a HLA-matched donor is not available for XL-CGD patients, paternal haplo-HSCT using TCR alpha/beta^+^/CD19^+^ depletion is an option worth to consider. In case of mixed donor chimerism and imminent graft failure, DLI administerd early in the course post HSCT, may allow to rescue a graft.

## Data availability statement

The raw data supporting the conclusions of this article will be made available by the authors, without undue reservation.

## Ethics statement

Informed consent for haploHSCT was obtained from the patient’s parents in accordance with local and EBMT guidelines. Before submission of the manuscript, additional informed consent for publication was obtained from both parents.

## Author contributions

HB diagnosed CGD. AS, JHS and HB planed the HSCT. JS, JHS and HB planned the manuscript. JS, AK, AS, FZ, LM, JHS and HB cared “hands on” for the patient, prior and post HSCT. AE and PL supervised HSCT. JS collected and analyzed the data, wrote the initial version of the manuscript and made figures and tables. HB finalized the manuscript. All authors read and approved the manuscript. All authors agree to be accountable for the content of this work.

## Glossary

**Table:**

ANC	absolute neutrophil counts
ADV	adenovirus
allogeneic HSCT	hematopoietic stem cell transplantation
AUC	area under the curve
ATG	anti-thymocyte globulin
AR-CGD	autosomal recessive CGD
CGD	chronic granulomatous disease
CMV	cytomegalovirus
DLI	donor lymphocyte infusion
DHR	dihydrorhodamine
EBMT	European Society for Blood and Marrow Transplantation
EBV	Epstein Barr virus
GVHD	graft versus host disease
GCSF	granulocyte colony stimulating factor
HLA	human leukocyte antigen
haplo-HSCT	HLA-haploidentical HSCT
HSCT	hematopoietic stem cell transplantation
IEI	inborn error of immunity
INF- γ	interferon- γ
MMF	mycophenolate
MAC	myeloablative conditioning
NIH	National Institutes of Health
NADPH	nicotinamide adenine dinucleotide phosphate
OS	overall survival
PCR	polymerase chain reaction
PBMC	peripheral blood stem cells
RIC	reduced intensity conditioning
RTC	reduced toxicity conditioning
TCR	T-cell receptor
TMP-SMX	trimethoprim-sulfamethoxazole
VOD/SOS	veno-occlusive disease/sinusoidal obstruction syndrome
XL-CGD	X-linked chronic granulomatous disease
