# Supplementary material for: Case report: HLA-haploidentical HSCT rescued with donor lymphocytes infusions in a patient with X-linked chronic granulomatous disease
Source: Front Immunol. 2023 Feb 16;14:1042650. doi: 10.3389/fimmu.2023.1042650 (PMC9978143; doi:10.3389/fimmu.2023.1042650)
Supplement: Supplementary file 1 [file Table_1.pdf]

Supplementary Table 1: Haploidentical HSCT in CGD between 2006 and 2022.

Publication	Year	CGD Patients (age)	Neutrophil Engraftment (day, median)	Graft Failure (day)	Graft	Conditioning Regimen	GVHD Prophylaxis	GVHD (grade, organs)	Reference
Kikuta et al.	2006	1 (2 y/a)	15	1 (day 77)	T cell depleted PBSC	RIC (FLU/MEL/ATG)	Tacrolimus MTX Prednisolone	no	(16)
Hoenig et al.	2014	1 (6 y/a)	16	-	CD34+ selected PBMC	MAC (BU/FLU/TT/A)	no	no	(17)
Parta et al.	2015	1 (12 y/a)	24	-	unmanipulated PBC	RTC (FLU/BU/CY)	Cyclophosphamide Sirolimus	Grade II	(18)
Morillo-Gutierrez et al.	2016	1 (3.6 y/a)	16	-	TCRαβ$^+$ depleted PBSC	RTC (TREO/FLU/TT/A)	Cyclosporin A	Grade I (skin)	(19)
Klein et al.	2016	1 (5 y/a)	19	-	unmanipulated bone marrow	RTC (FLU/MEL/TBI/A)	Cyclophosphamide Mycophenolate mofetil Tacrolimus	Grade I (skin)	(20)
Zhou et al.	2017	1 (13 y/a)	23	-	unmanipulated bone marrow (day +1) PBMS (day +2)	MAC (CYT/BU/CY/ATG)	Cyclosporin A MTX Mycophenolate mofetil	Grade II (skin)	(21)
Shah et al.	2018	2 (2.4; 3.7 y/a)	15	-	TCRαβ$^+$/CD19$^+$ depleted PBSC	MAC (TREO/FLU/TT/A) (TREO/FLU/TT/rATG)	Cyclosporin A Mycophenolate mofetil	Grade I-II (skin)	(22)
Regueiro-Garcia et al.	2018	1 (11 y/a)	17	-	unmanipulated PBSC	MAC (TREO/FLU/rATG)	Cyclophosphamide	no	(23)
Lum et al.	2019	4 (median 5.3 y/a)	16	-	TCRαβ$^+$/CD19$^+$ depleted PBSC	MAC (BU/CY) (FLU/TREO/A)	Cyclosporin A Mycophenolate mofetil	Grade II-III	(24)
Brettig et al.	2019	2 (17,2; 3.9 y/a)	12	-	TCRαβ$^+$/CD19$^+$ depleted PBSC	MAC (TREO/FLU/TT)	Cyclosporin A Mycophenolate mofetil	no	(25)
Neven et al.	2019	1 (7.7 y/a)	19	-	unmanipulated bone marrow	MAC (FLU/BU/A/rATG)	Cyclophosphamide	Grade III	(26)
Mitchell et al.	2019	1 (NA)	10	1 (day 21)	TCRαβ$^+$/CD19$^+$ depleted PBSC	MAC (TREO/FLU/TT)	Mycophenolate mofetil	NA	(27)
Parta et al.	2020	3 (14-16 y/a)	19	-	unmanipulated PBSC	RTC (CY/FLU/TBI/BU)	Sirolimus	Grade ≥ II	(28)
Holzer et al.	2021	1 (3.13 y/a)	10	-	TCRαβ$^+$ depleted PBSC	MAC (FLU/TREO/TT/ATG)	NA	no	(29)
Merli et al.	2022	1 (NA)	NA	-	TCRαβ$^+$/CD19$^+$ depleted PBSC	(ATG/RIT)	no	NA	(30)

Lum et al.	2022	2 (median 3.28 y/a)	14	-	TCRαβ+/CD19+ depleted PBSC	MAC (TREO/FLU/TT/rATG/RIT)	no	no	(31)

Abbreviations:
Alemtuzumab, A; busulfan, BU; cyclophosphamide, CY; Cytarbine, CYT; fludarabine, FLU; methotrexate, MTX; not available, NA; peripheral blood stem cells, PBSC; rabbit anti-T lymphocyte globulin, rATG; rituximab, RIT; reduced toxicity conditioning, RTC; T cell receptor, TCR; total body radiation, TBI; treosulfan, TREO; thiotepa, TT
